# Developing a bioinformatics pipeline for comparative protein classification analysis

**DOI:** 10.1186/s12863-022-01045-x

**Published:** 2022-06-06

**Authors:** Benedetta Pelosi

**Affiliations:** Department of Molecular Biosciences, The Wenner-Gren Institute, Stockholm University, Stockholm, Sweden

**Keywords:** Biosynthetic pathway, Carotenoid biosynthetic genes, Comparative genomics, *Brassica rapa*, *Brassica rapa* Pekinensis group, *Arabidopsis thaliana*, Bioinformatics pipeline

## Abstract

**Background:**

Protein classification is a task of paramount importance in various fields of biology. Despite the great momentum of modern implementation of protein classification, machine learning techniques such as Random Forest and Neural Network could not always be used for several reasons: data collection, unbalanced classification or labelling of the data.As an alternative, I propose the use of a bioinformatics pipeline to search for and classify information from protein databases. Hence, to evaluate the efficiency and accuracy of the pipeline, I focused on the carotenoid biosynthetic genes and developed a filtering approach to retrieve orthologs clusters in two well-studied plants that belong to the Brassicaceae family: *Arabidopsis thaliana* and *Brassica rapa* Pekinensis group. The result obtained has been compared with previous studies on carotenoid biosynthetic genes in *B. rapa* where phylogenetic analysis was conducted.

**Results:**

The developed bioinformatics pipeline relies on commercial software and multiple databeses including the use of phylogeny, Gene Ontology terms (GOs) and Protein Families (Pfams) at a protein level. Furthermore, the phylogeny is coupled with “population analysis” to evaluate the potential orthologs. All the steps taken together give a final table of potential orthologs. The phylogenetic tree gives a result of 43 putative orthologs conserved in *B. rapa* Pekinensis group. Different *A. thaliana* proteins have more than one syntenic ortholog as also shown in a previous finding (Li et al., BMC Genomics 16(1):1–11, 2015).

**Conclusions:**

This study demonstrates that, when the biological features of proteins of interest are not specific, I can rely on a computational approach in filtering steps for classification purposes. The comparison of the results obtained here for the carotenoid biosynthetic genes with previous research confirmed the accuracy of the developed pipeline which can therefore be applied for filtering different types of datasets.

**Supplementary Information:**

The online version contains supplementary material available at (10.1186/s12863-022-01045-x).

## Background

Classifying protein sequences is widely used to predict the structure and function of newly discovered proteins. However, many existing computational techniques can give unreliable results due to a broad size of features: especially with finite amounts of labeled source data, these heuristic techniques can induce significant estimation errors in settings with large sample selection bias [1].

Filtering libraries for classifying proteins is still an open challenge in molecular biology: type of data collection, unbalanced classification and labeling of the data are all parameters that a researcher needs to take in consideration [2–6]. To address the above mentioned issues, I propose an “Occam’s razor” filtering method to achieve a model biased to the simplest function that fits the data. To understand the structural, functional, and evolutionary relationships among the proteins of interest, I developed a pipeline – a flow of computational proceedings – which includes the use of Gene Ontology (GO) terms [7], Protein families (Pfam) (functional/ structural features analysis) [8] and multiple phylogenetic analysis (deterministic filtering coupled with different biases analysis). I tested the filtering bioinformatics pipeline on a class of isoprenoids, carotenoids, in two well-studied plants that belong to Brassicaceae family: *Arabidopsis thaliana* and *Brassica rapa* Pekinensis group.

**Fig. 1:**
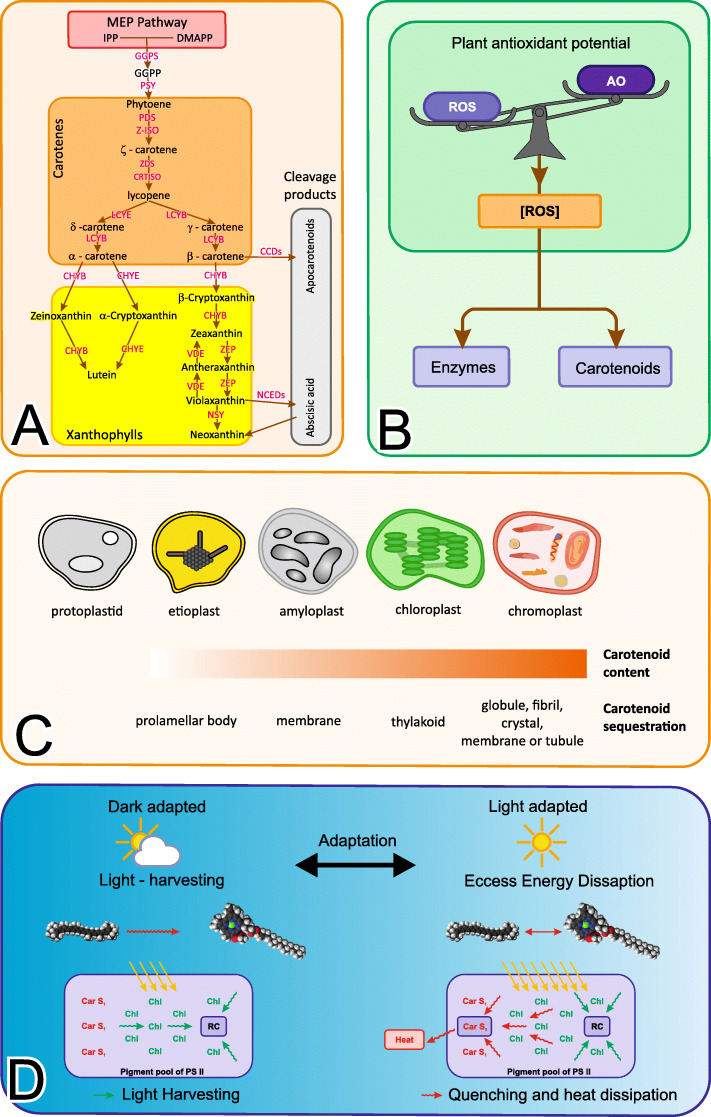
Carotenoids play major roles in plants as antioxidants, accessory light-harvesting pigments. As an effect of increased ROS production, the activity and biosynthesis of different plant antioxidants raise up in response for preventing oxidative damage. A novel function for carotenoids relates to the response of plants to abiotic stress factors as shown (left). A compressed version of the carotenoid biosynthesis pathway is shown on the right: Isopentenyl pyrophosphate (IPP) – biosynthesis, Phytoene biosynthesis, Lycopene biosynthesis, Lycopene cyclization, Sequestration and storage [A]. Carotenoid metabolism in light of plastid types in plants and sequestration in relation to these metabolites accumulation are shown in [B].The photoprotective role of these metabolites derives from their ability to quench excited chlorophyll states, scavenge ROS and dissipate excess energy as heat during the light adapted state (Fig.C). The electronic interactions between carotenoid dark states and chlorophylls are shown on the right. If the energy levels of Car S1 and Chl aQy are similar,increased electronic interactions cause to the formation of excitonic states which are delocalized [112]. Part of the figure is adapted from [10]

The two major classes of carotenoids are: carotenes (hydrocarbons that can be cyclized at one or both ends of the molecule) and xanthophylls (oxygenated derivatives of carotenes) (Fig. 1**A, B**) [9]. Carotenoids usually accumulate in the chromoplasts which sequester large amounts of carotenoids in plastoglobules or/and in storage structures of several shapes made of lipids and proteins. All other plastid types can synthesize carotenoids, but the level of accumulation varies broadly among different plastid types (Fig. 1**C**) [10, 11]. Carotenoids are also found in the chloroplasts of photosynthetic tissues and mainly together with chlorophylls, in functional pigment-binding proteins embedded in photosynthetic (thylakoid) membranes [12]. One of the primary roles of carotenoids is to protect the photosynthetic apparatus by quenching of chlorophyll triplets and singlet oxygen, and dissipating the excess light energy by nonphotochemical quenching of chlorophyll fluorescence [13]. In details, these metabolites play a role in photosynthetic light energy capture, conversion, and reduction of Reactive Oxygen Species (ROS) dissipation, due to fast thermalization (Fig. 1**B, D**). In this work, I mainly focus on the photoprotective function (AntiOxidant(AO)) of these metabolites in the oxygenic photosynthesis for classifying the carotenoid elements.

*A. thaliana* has been useful for studying of the core carotenoid biosynthetic pathway and its regulation due to the extensive information about candidate *A. thaliana* genes and enzymes involved in the biosynthesis of isoprenoids [14]. Nowadays, I have an almost complete picture of the carotenoid biosynthetic pathways in *A. thaliana* [14]. Here, carotenoids are synthesized from the five carbon units isopentenyl diphosphate (IPP) and its double-bond isomer dimethylallyl diphosphate (DMAPP) [15] produced by the plastidial 2-C-methyl-Derythritol 4-phosphate (MEP) pathway, as shown in Fig. 1**A**.

The genus Brassica includes, among others, *B. rapa*, *B. oleracea*, *B. napus*, *B. parachinensis*, and *B. juncea* which are the most economically important [16, 17]. Furthermore, a number of 11 subspecies of *B. rapa* were divided into two distinctly different groups, namely Pekinensis (pe-tsai group, the more common) and Chinensis (pak choi group) [18]. Little is known about the genes in the carotenoid biosynthetic pathway of genus Brassica, thus *B. rapa* Pekinensis group has been object of research studies, in order to clarify such mechanism [19]. To validate the performance of the proposed pipeline in protein classification, I decided to focus my analysis on the carotenoid biogenesis of *B. rapa* Pekinensis group, a group of wildly cultivated vegetables also referred in literature as Chinese cabbage or *B. rapa* subsp. *pekinesis* or *B. rapa campestris* [20–23] (https://gd.eppo.int/taxon/BRSPK). This species has important anti-oxidative features that are currently being developed to improve the quality of vegetables and hence human health [24–27]. The Pekinensis group of *B. rapa* is called with various names (https://gd.eppo.int/taxon/BRSPK) which sometimes overlap with the Chinensis group. In this work I analyzed the pe-tsai group and, to avoid confusion, I will hereinafter refer to it as *B. rapa* Pekinensis group. The first reference genome study of *B. rapa* Pekinensis group was released in 2011 [28]. Since then, *B. rapa* Pekinensis group has become an attractive model system for plant growth modeling because of its close relationship with *A. thaliana*.

To confirm the validity of my pipeline by obtaining comprehensive information on the carotenoid biosynthetic pathway in *B. rapa* Pekinensis group, I performed a protein classification analysis between *A. thaliana* and the *B. rapa* Pekinensis group using the sequences and annotation information of the two species [29, 30]. Since carotenes and xanthophylls can be modified to create the broad diversity of carotenoids found in plants and other organisms, I included different “putative proteins-outgroups” in the last part of phylogenetic analysis in order to better define the homologous clusters in the MEP pathway. Carotenoid diversity is much significant for its biotechnological potential [31, 32] and its role played in understanding the evolution of secondary metabolism.

I also chose for my study two microorganisms of interest that I used as outgroup in phylogenetic studies. One of them, the microalgae *Chlamydomonas reinhardtii* [33, 34] has evolved different types of carotenoids since this class of pigments are used as precursors of various other molecules with pivotal physiological functions in the species [33, 35, 36]. Furthermore, synthesis and regulation of the carotenoid biosynthetic genes is shown to be triggered and regulated by different stress responses [37, 38]. Remarkably, the increase of carotenoids can work as a protection mechanism in cryospheric environments for psychrophilic bacteria [39]. Therefore, as an outgroup, I also introduced *Hymenobacter psychrophilus*, a cold-loving gram-negative bacterium isolated from soil in an industrial site in Bolzano (South Tyrol, Italy). This species was chosen because of the unpredictable distribution of carotenoids proposed for the genus *Hymenobacteras* [40] as consequence of various events of gene gain, gene loss, or evolution of regulatory mechanisms in this genus. In particular, I chose to focus on Brp/Blh, a putative *β*-carotene diooxygenase from *H. psychrophilus* (https://www.ebi.ac.uk/ena/browser/view/PRJEB16609) that may be well conserved in other species of *Hymenobacter* [41, 42]. Furthermore, I included as an outgroup *A. thaliana* cytochrome P450 since monooxygenases are known to play a role in the biosynthesis of various compounds [43, 44].

Here, I report a systematic analysis of proteins involved in carotenoid biosynthesis in the Pekinensis group. The analysis has identified 43 putative orthologs. Moreover, I have evaluated the accuracy of my bioinformatics pipeline on proteomics datasets available on public databases. This type of study is particularly valuable for validating a filtering approach that is useful for data classification when sampling bias cannot be assessed.

## Results

### Filtering steps

**Fig. 2:**
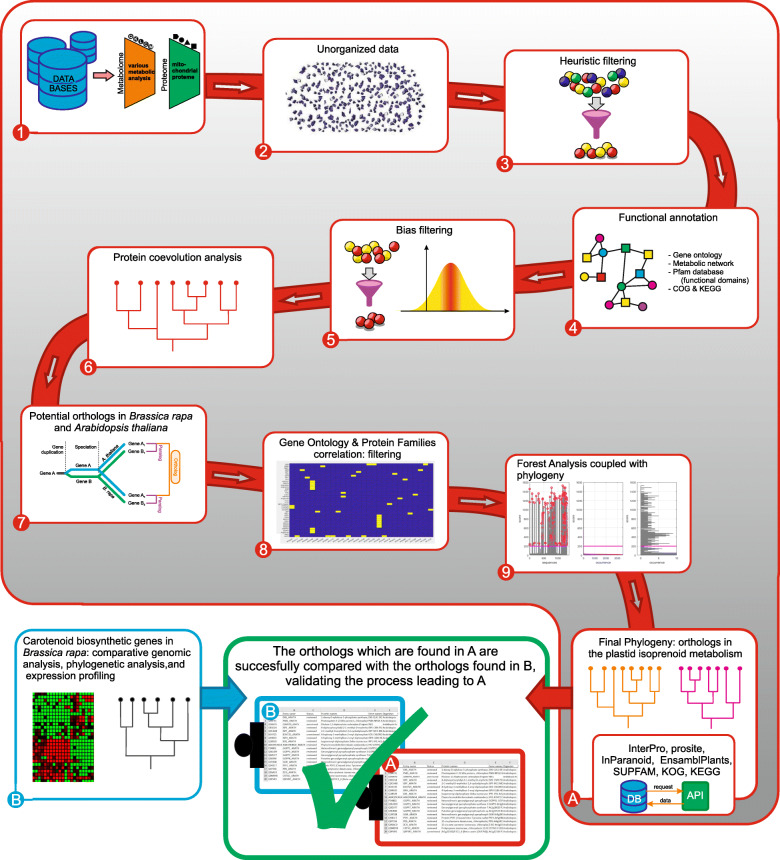
Flow of filter of the bioinformatics pipeline. 1) Database extrapolation of data related to *A. thaliana* and *B. rapa* Pekinensis group 2) Unorganized raw data 3) Heuristic Filtering scripts 4) Functional annotation retrieved from Uniprot Swiss/TrEMBL 5) Bias Filtering section 6) “Raw” phylogenetic analysis 7) potential homologous selection 8) Gene ontology/Pfam screening association for functional domains 9) Population analysis simulated – statistics score applied; reference sequences from *A. thaliana* TAIR database a) phylogeny to analyze the population analysis result coupled with the previous raw phylogenetic analysis results; new databases extrapolation of input data through API and specific phylogenetic analysis of MEP pathway gene products b) comparison of my pipeline with previous findings in *B. rapa*

A schematic representation of the bioinformatics pipeline developed in the present study is presented in Fig. 2. In the first step of the pipeline, and in order to retrieve all the proteins of *A. thaliana* that are annotated as being related to chloroplasts and/or plastids in public databases, I used the string searches “chloroplast proteome”, “thylakoid proteome” and “carotenoids” in UniProtKB and UniParc. I mainly focused on the aforementioned taxa in relation to the role of carotenoids in the oxygenic photosynthesis and in oxidative reactions involved in ROS/AO balance (Fig. 1). This search resulted in 5222 proteins. This large number originates from the fact that a great number of proteins is reported from the fusion of two databases (UniProtKB composed of Swiss-Prot and TrEMBL sections linked to UniParc proteomics) in which some protein names are doubled under not univocal entry name – leading to “database redundancy” – and some entry are not specifically classified. All the data retrieved (5222 elements) were merged and a number of filtering steps were devised to identify putative carotenoid elements.

First, the organism specific proteins for the plant species of interest were selected. Following, to shortlist the proteins involved in photosynthesis, photo-protection, oxidative stress, plant coloration and cell signaling, I examined the gene ontology terms with a particular focus on “gene ontology molecular function” and “gene ontology biological process” (see Additional file 13: Table 1 and see the MATLAB code in Additional file 9).

**Fig. 3:**
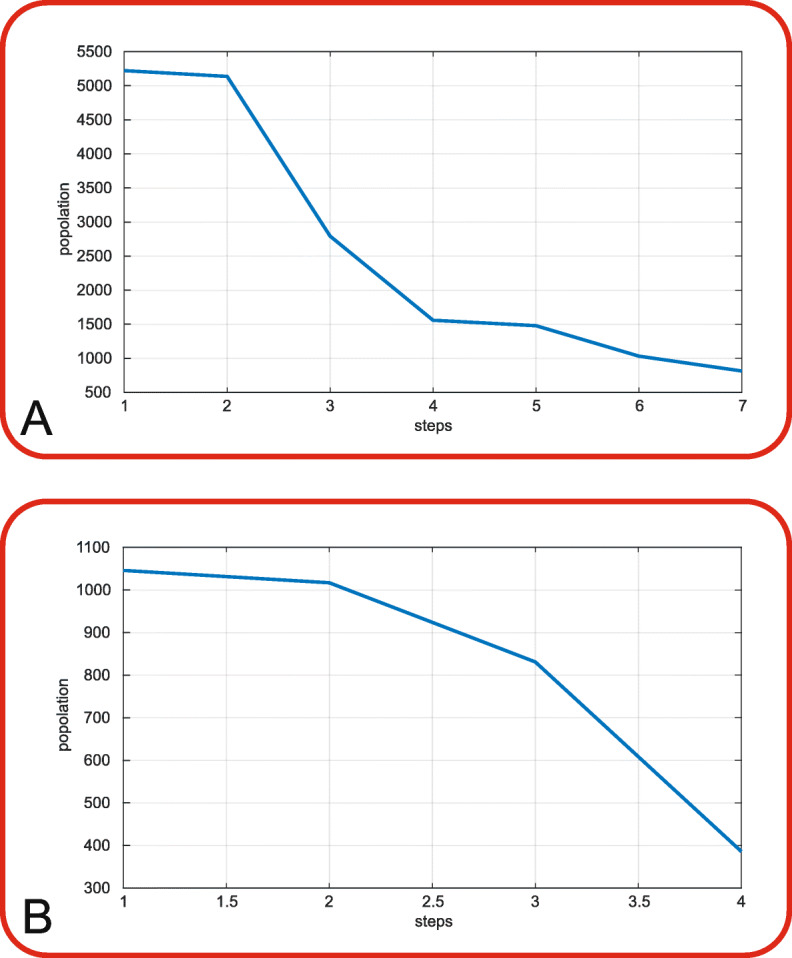
Filtering steps in *A. thaliana* and *B. rapa* Pekinensis group [in x-axis the steps of the filter, in y-axis the population corresponding to each step]. The plot **A** shows the trend of the filter population in *A. thaliana*. Starting from 5222, the population is filtered in 8 steps till reaching 813 elements: 5222, 5137, 2793, 1558, 1478, 1031, 813. The plot **B** shows the trend of the filter population in *B. rapa Pekinensis group*. From 1046, the population is filtered in three steps till reaching 386 elements. I obtained 1046, 1017, 831, 386 proteins

Next I retrieved further information by means of Pfam classification databases, Protein Families and Panther [45] to filter the 5222 elements identified above. The code strings processed the proteins by a continuous filtering analysis based on Pfam and GOs for making a reduction of the *A. thaliana* proteome. Starting from a number of 5222, I obtained 5137, 2793, 1558, 1478, 1031, 813 proteins in six steps (Fig. 3**A**, see Additional file 13: Table 1). The same analysis was repeated for Pekinensis group: starting from 1046 proteins, I obtained 1017, 831, 386 in three steps Fig. 3**B**, see Additional file 14: Table 2).

### Inferred phylogeny

To firmly establish a link between the elements identified above and the carotenoid biosynthetic pathway, I carried out a phylogenetic analysis and looked for putative potential orthologs. The 1199 proteins obtained from the analysis above – 813 in *A. thaliana* and 386 in Pekinensis Group – were screened for duplicates/redundancy, which resulted into 1089 unique entries that were used for the phylogenetic analysis.

I chose to use the commercial software MEGAX [46] (see Methods) for inferring phylogenetic relationships since it is more accurate for evaluating clusters among different input-proteins lengths [47, 48] within specific species.

The tree showed the clusters and allowed to identify the putative conserved elements. The analysis was performed 100 times: with reference to Additional file 1, the bootstrap is shown in the tree as the number of events in which that particular cluster has been reached. This gives a probabilistic demonstration high protein conservation and the results were significant in a number of more than 75 times [46].

However, I considered bootstrap values between 11 and 50 as acceptable putative homologous since there are errors caused by the “multiple noise interferences” in the clustering due to the effect of a broad sampling of different protein sequences lengths – “weights”. Eventually, a number of 180 proteins in *B. rapa* Pekinensis group were noted in a table as potential chloroplast carotenoid orthologs (Additional file 15: Table 3, see also Additional file 2).

### GO terms of putative carotenoid orthologs

The list of 180 potential orthologs resulted from the phylogenetic analysis was studied for GO terms enriched groups cataloged for molecular function, since the previous deterministic filter was affected by some noise due to the broad amount of data analyzed. Among the GO terms found to be associated with the 180 putative orthologs, the following were related to oxygenic photosynthesis: carotenoid dioxygenase activity [GO:0010436], oxidoreductase activity [GO:0016730], metal ion binding [GO:0046872], deoxy-D-xylulose-5-phosphate synthase activity, [GO:0016744], transferase activity, transferring aldehyde or ketonic group, GO:0102067- geranylgeranyl diphosphate reductase activity, carotenoid isomerase activity [GO:0046608], oxidoreductase activity [GO:0052887], farnesyl-diphosphate farnesyltransferase activity [GO:0004310]. The 180 elements were additionally analyzed according to the Crossreference Pfam for checking the functional domains by means of Hidden Markov Models when available (Additional file 15: Table 3) [8, 49].

**Fig. 4:**
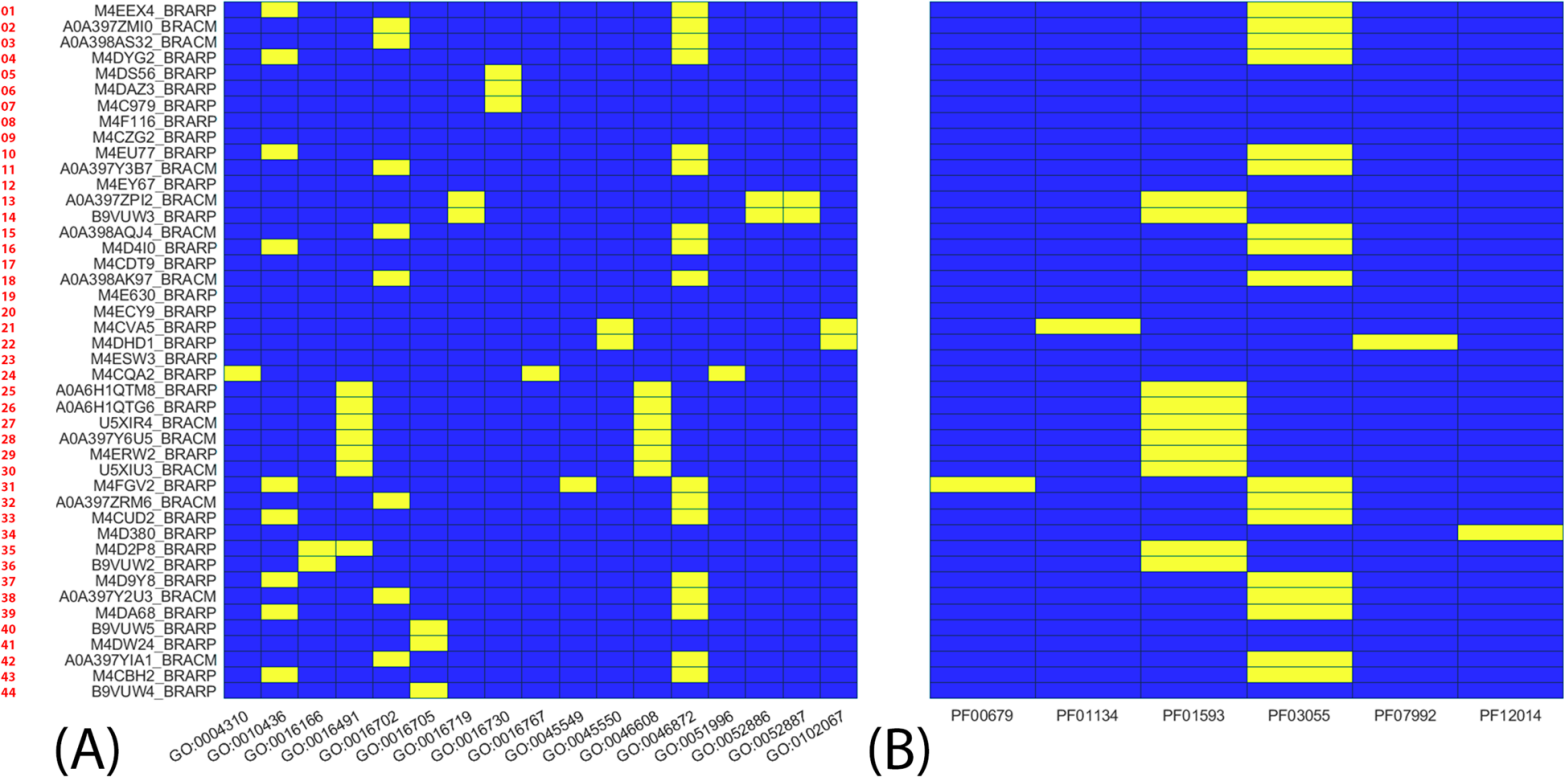
GO terms and Pfam domains in the 44 identified orthologs. The heat maps visualize the metabolites involved in the oxygenic photosynthesis. The 44 elements are indicated in the y axis. GO terms (**A**) and Pfam domains (**B**) are in the x axis. BRARP = *Brassica rapa* subsp. *pekinensis*, BRACM = *Brassica rapa campestris* var. *pekinensis*. Both BRARP and BRACM are cataloged as *B. rapa* Pekinensis group. The association of a specific GO term or Pfam domain with each of the proteins is indicated in yellow

The following Pfam domains were found: amino_oxidase (PF01593) and carotenoid oxygenase (PF03055), the two ones associated with many elements. Furthermore, Pyr_redox_2 (PF07992) includes families of oxyreductase and it was associated with one protein element. Based on the GO and Pfam analysis described above, the orthologs that did not play a role in oxygenic photosynthesis, labeled in Additional file 15: Table 3 in yellow, were discarded, which resulted in 44 potential orthologs as the result of my analysis (see Additional file 16: Table 4). The association between each protein ID and GO term or Pfam term is represented by the means of two heatmaps, i.e. two color-coded matrices where blue and yellow represent the absence or presence of a specific GO for a given protein ID, respectively (Fig. 4, see also Additional file 2, in orange the removed elements; see also Additional file 10 for the MATLAB code).

### Forest analysis coupled with phylogenetic analysis

As mentioned above, when using the Uniprot database as a starting source, 44 proteins in *B. rapa* Pekinensis group were identified as highly conserved putative chloroplast protein orthologs involved in carotenoid biosynthesis.

Next, a “population” analysis has been applied: here and hereinafter the use of the term “population” refers to “all related protein from a species”. I wanted to apply a population analysis by using reviewed sequences obtained from The *Arabidopsis* Information Resource (TAIR)[50]. For this purpose, I used the total Pekinesis group chloroplast filtered population in order to help the localization of all the pathways that produce isoprenoids. A number of 386 elements were used, as result of the filtering steps section.

The population analysis retrieved a variety of 47 characterized proteins when using “carotenoid biosynthetic process” as a string query (reviewed on TAIR [50]). These 47 proteins play a role in the MEP pathway and in the oxygenic photosynthesis. Therefore, I had a total of 47 potential *A. thaliana* orthologs (see Additional file 17: Table 5) that I use as reference sequences to obtain a much more “discriminated” situation already with the single samples. The 47 sequences were aligned versus the chloroplast proteins and carotenoid factors retrieved from Additional file 14: Table 2.

**Fig. 5:**
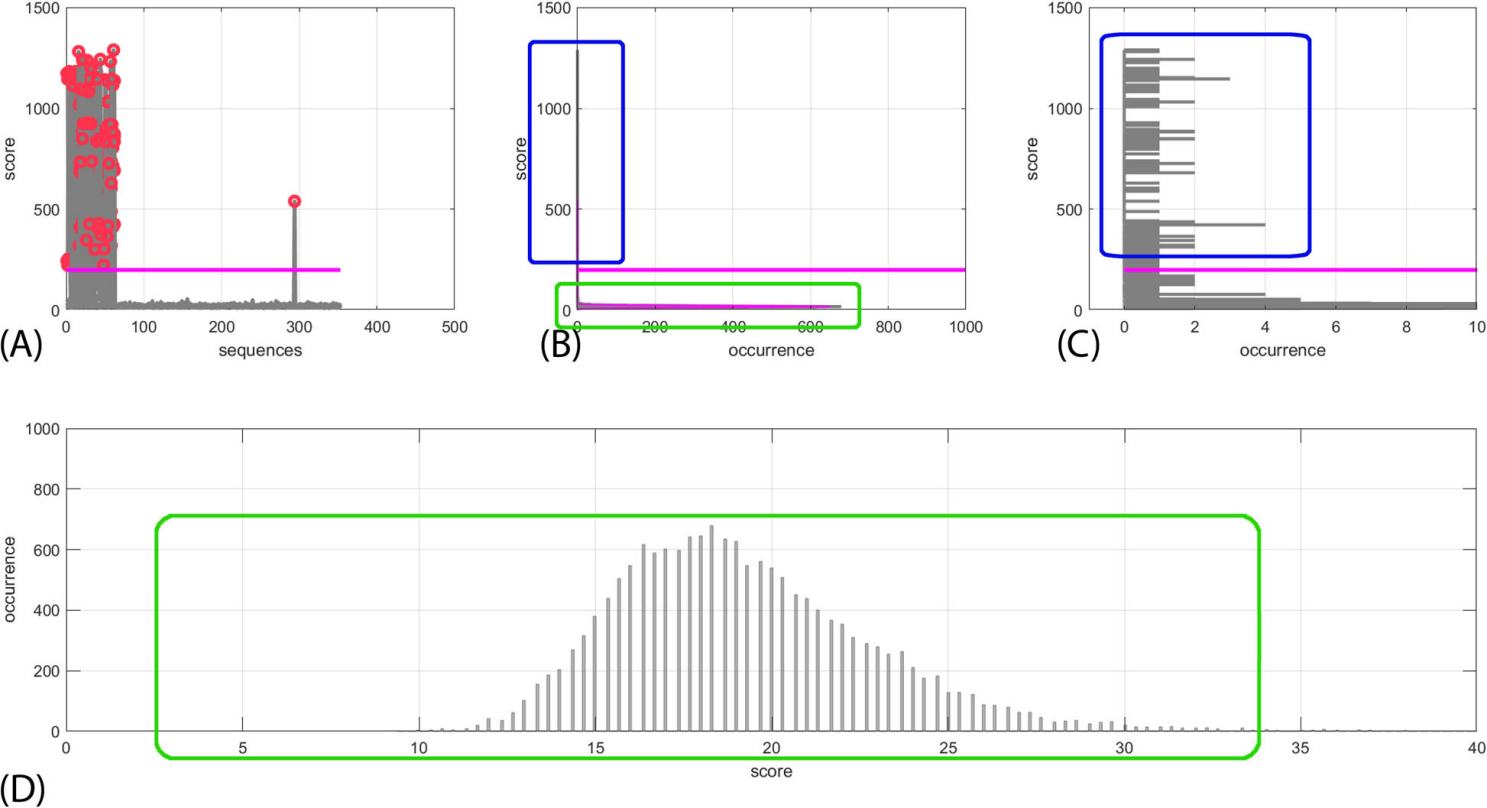
Forest histogram and bimodal distribution. Forest (**A**). The forest is computed: here sequences that correlate more are shown with circle. The circle pointed out the score which was selected for higher correlation between references sequences and population. Sequences exceeded a prefixed value, fixed at 200 in this case, is highlighted. Bimodal distribution (**B**). The statistics of the score distribution is shown to explain the score used. It is divided in two zone: a lower zone, highlighted in green, and a upper zone, highlighted in blue. Detail of the upper zone of the bimodal distribution (**C**): the blue zone of (**B**) is zoomed out in x. In the upper zone, only the sequences which posses a strong correlation with the references are present. Their distribution, highlighted in blue, is almost uniform. Detail of the lower zone of the bimodal distribution (**D**): the green zone of (**B**) is zoomed out in y and rotated. In the lower zone, the distribution highlighted in green, resembles a Gaussian distribution. This is expected for a merely random correlation

The resulting values of each input population against the references are plotted in a 2D graph, depicted in Fig. 5 (see Additional file 11 for the MATLAB code). Each sequence whose score exceeds a prefixed value, 200 in this case, is shown: this value is motivated by the following plot (Fig. 5**A**), where I showed the statistics of the score distribution. The statistic “score” reveals a “bimodal distribution” form (Fig. 5**B, C**), with a lower circumscribed region, corresponding to the many cases in which the sequences correlate for a short duration with the samples.

This zone, zoomed in Fig. 5**D**, resembles a Gaussian distribution, as expected for a merely random correlation. An upper zone follows, much more diluted, of various levels of correlation, resembling a uniform distribution: 62 sequences were finally obtained with high correlation (Fig. 5**C**). Many sequences totalize a very low score, meaning that it is reasonable that a low scoring value is due to random match of many sub-sequences. The comparison of the forest and the statistic is emphasized: the value around 200 is a good compromise for the beginning of the area of strong correlation.

**Fig. 6:**
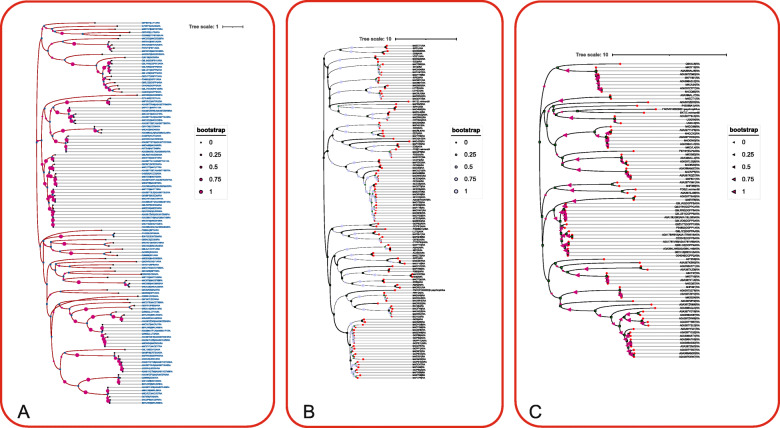
Phylogenetic analysis derived from Forest Analysis and GO coupled with Pfam terms screening. **A**: The tree with the highest log likelihood (-38190.90) is shown. The associated taxa clustered together next to the branches are defined by a normalized scale. The bootstrap is highlighted by pink circles (normalized scale from 0 to 1). The internal nodes are represented by rectangular shape in blue and each protein ID is highlighted with dashed lines. The leaf nodes are displayed with a circular shape. ARA = *A. thaliana*, RA = *B. rapa* Pekinensis group are the labels to refer to the species. **B** The tree with the highest log likelihood (-71352.70) is shown. The tree is drawn to scale, with branch lengths measured in the number of substitutions per site. The bootstrap is highlighted by purple circles (normalized scale from 0 to 1). The internal nodes are represented by rectangular shape in green and each protein ID is highlighted with dashed lines. The leaf nodes are displayed with a rectangular shape in red. ARA = *A. thaliana*, RA = *B. rapa* Pekinensis group. Four outgroups are also inclided in the tree: Bkt from *C. reinhardtii*, Pds *C. reinhardtii*, FNOV0100002 - Brt/brk from *H. psycrophilus*, P45086A1 - cytochrome P450 from *A. thaliana*. **C** The tree with the highest log likelihood (-41791.08) is presented. The bootstrap is highlighted by purple triangles (normalized scale from 0 to 1). The internal nodes are represented by rectangular shape in green and each protein ID is highlighted with dashed lines. The leaf nodes are displayed with a circular shape in red. The four outgroups are included as in the previous analysis. ARA = *A. thaliana*, RA = *B. rapa* Pekinensis group are the labels to refer to the species

The forest analysis results of 62 *B. rapa* Pekinensis group elements (Additional file 3) and the 44 *B. rapa* Pekinensis group sequences obtained from the first phylogenetic analysis coupled with the GO/Pfam heat map screening (Additional file 16: Table 4) were added to the 47 reference sequences from *A. thaliana* retrieved from TAIR (Additional file 17: Table 5). Next, a new phylogeny (Fig. 6**A**) was inferred. The final phylogeny gave a result of 40 potential orthologs between *B. rapa* Pekinensis group and *A. thaliana* (Additional file 18: Table 6). The elements of the MEP pathway and the *GGPS* isoforms have not been fully cataloged in the Pekinensis group [51, 52]. To this end, I computed two phylogenetic trees by taking the elements belonging to the MEP pathway and the *GGPS* isoforms from the previous phylogenetic analysis. Furthermore, I took into account that different *A. thaliana* carotenoid factors could have more than one syntenic ortholog in *B. rapa* Pekinensis group species [53].

To attempt to retrieve all the potential syntenic and non-syntenic orthologs in *B. rapa* Pekinensis group, *A. thaliana* “reference” carotenoid sequences and *B. rapa* Pekinensis group potential carotenoid elements were retrieved by using string-searches in Uniprot database, EsemblePlants, Inparanoid, Prosite, InterPro, KOG, SUPFAM and STRING APIs linked to Uniprot [54–61].

A number of 35 elements from *B. rapa* Pekinensis group were retrieved from STRING, KOG, Inparanoid, EggNOG, Pfam and SUPFAM using the string queries “oxidoreductase” and “reductase” (see Additional file 4). These 35 were added to the 40 elements from *B. rapa* Pekinensis group obtained from the forest coupled with phylogeny. Following the 75 *B. rapa* Pekinensis group elements were added to the 49 *A. thaliana* sequences (see Additional file 5) of putative carotenoid elements involved in the MEP pathway retrieved from Inparanoid, EsemblPlants, RefSeq [62], InterPro via string queries “carotenoid” and “isoprenoids”.

Four outgroups were included in the analysis. Outgroups were selected in different organisms where Carotenoids have an important role in the protection of the photosynthetic apparatus, but there are differences in the distribution or regulation of these metabolites [31, 34, 38, 43, 63–66].

The outgroup *C. reinhardtii* and *H. psychrophilus* were used to improve the clustering method of the cladograms for better estimating the divergence [48, 67, 68].

*C reinhardtii* was used since various carotenoids play important roles in response to abiotic stress conditions [36–38]. Additionally, *H. psychrophilus*, a psychrophilic bacterium, has been introduced as outgroup since the evolution of carotenoid biosynthetic genes have occurred in a different way, probably due to some adoptive mechanisms in cryospheric environment [31, 39]. I finally selected the hemoprotein cytochrome P450 from *A. thaliana* as an outgroup since it is not related to carotenoids. Finally, a phylogenetic analysis was inferred to a total of 128 elements. This analysis (Fig. 6**B**) was coupled with a phylogenetic analysis to highlight the *GGPS* elements conserved in *B. rapa* Pekinensis group.

Carotenoid putative gene products were retrieved by using the string search of “isoprenoids” for a total number of 37 *B. rapa* Pekinensis group KOG, InParanoid and EnsemblPlant elements (Additional file 6) and 27 *B. rapa* Pekinensis group elements via queries “carotenoid oxygenase” (Additional file 7) from InParanoid, InterPro and EMBL [69]. A number of 17 carotenoid elements (Additional file 8) were retrieved from Prosite, InParanoid, SUPFAM, EMBL and EggNOG by using the string search “GGPS” for *A. thaliana*.

The four outgroups used in the MEP pathway tree were also added to the list above in order to infer a more specific phylogenetic clustering computational method. The final dataset of 85 elements was subjected to phylogenetic analysis and the evolutionary tree is presented in Fig. 6**A**. Taken together the results presented in Fig. 6**B** and **C**, I finally reported my putative orthologs in Additional file 19: Table 7. *GGPS6*, *GGPS9*, *GGPS12* and *LUT1* from *A. thaliana* were not found in *B. rapa* Pekinensis group and confirmed the results on carotenoids studies [53]. *GGPS4* and *GGPS7* from *A. thaliana* correspond to Bra038544 in *B. rapa* Pekinensis group. Here, “putative synteny” is assumed as “high homology” which is shown in the cluster at the level of phylogenetic branch length and node of the tree (Fig. 6**C**). A number of 43 putative orthologs were noted as a final result by combining the Forest analysis coupled with phylogeny and the phylogenetic analysis of the MEP pathway enzymes. A summarized flowchart of the whole process is shown in Additional file 12.

With reference to Additional file 19: Table 7 the non syntenic ortholog shared by *GGPS1*, *GGPS2*, *GGPS3*, *GGPS7*, *GGPS8* was not found (highlighted in green in table), whereas I retrieved some potential orthologs for *DXS* (Bra001832), *GPS11* (cag7890226(Bra)) and *BCH2* (Bra008358) (in bold and highlighted in blue in Additional file 19: Table 7) which need further verification (e.g. syntenic analysis [70–73]). Interestingly, at least *GPS11* showed to have an ortholog by doing a Blast search on NCBI and Ensembl Plant (Blastp not shown). Furthermore, I also identified a gene product probably related to carotenoid elements (highlighted in orange in table): *PSBS*-like gene (Bra036950). In conclusion, a sort of error occurs, which can be estimated as the percentage of the orthologs not found in the reference work [53] with respect to all the orthologs retrieved, and can be calculated as 5.6%, computed as the number of the extra elements found (4, namely Bra001832, cag7890226, Bra008358, Bra036950) over the total number of orthologs counted in the table (72).

## Discussion

In this study, I performed a comparative protein classification analysis between *A. thaliana* and *B. rapa* Pekinensis group (i.e. the mustard family group called either *B. rapa* Pekinensis group or *B. rapa campestris* L.) using the protein sequences and annotation information of the two species [54]. I only focused on protein level, and not on nucleotide sequences, to better exploit the higher degree of conservation that exists in the amino acid sequences.

In order to validate the proposed pipeline, the ontology analysis of carotenoid biosynthetic gene products in *B. rapa* Pekinensis group was compared with results achieved in a previous study [53]. With this purpose, I developed a system of classification in steps – pipeline – for cataloging putative elements in the carotenoids pathways of *A. thaliana* and *B. rapa* Pekinensis group. Random Forest (RF) could not work for the purpose, because the biological properties of the proteins are not exact weights in terms of numeric values to develop a classifier and a neural network system [74].

Instead, I developed a computer-based analysis which classified a number of 1089 elements retrieved as chloroplastic proteins and reviewed enzymes of the carotenoid biosynthestic pathways. The functional analysis method that I propose makes use of GOs, Pfams, “population analysis” and phylogeny to identify the orthologs in *B. rapa* subsp. *pekinensis* using reference sequences from the well characterized model of the vascular plant *A. thaliana*. Previous studies showed that there are 67 carotenoid biosynthesis genes in *B. rapa* and 42 out of them have ambiguous orthologs (syntenic and not syntenic) in *A. thaliana* [53].

To assess the performance of my pipeline, I applied it to the well known carotenoid biosynthetic genes of *A. thaliana* and *B. rapa* Pekinensis group. As a first step, I retrieved the chloroplast and thylakoids proteomes of the two species and I filtered them by means of GO and Pfam terms for specific carotenoid oxidative function or linkage to the photosynthetic apparatus (“deterministic filtering process”) (Fig. 3). The high number of starting elements retrieved is due to the redundancy resulting from the fusion of Uniprot-Swiss/TrEMBL with UniParc proteomics [75]; it happens that one protein is referred with different IDs which carries to a not univocal identification of the same protein. This is a typical issue of not univocal annotations [76].

Following, I looked for the carotenoids conserved in the *A. thaliana* and *B. rapa* Pekinensis group. All the proteins obtained from the analysis above – 813 in *A. thaliana* and 386 in *B. rapa* Pekinensis group – were screened for redundancy via a script and, after the screening, 1089 total non-redundant elements were used for the next analysis. At this point, a phylogenetic analysis was inferred and the bootstrap method gave a first probabilistic demonstration of the protein conservation (Additional file 1).

A well established commercial software, [46], that has given satisfactory results in studies of inferred phylogeny, was employed. The use of this commercial software needs a preliminary alignment (MUSCLE-UPGMA or ClustalW [77]) of the sequences for estimating a preliminary covariance at the level of the substitution sites [78].

Eventually, a number of 180 gene products in *A. thaliana* had one ortholog in *B. rapa* Pekinensis group because I considered also bootstrap results between 11 and 80 for this preliminary analysis (Additional file 15: Table 3). The list of 180 potential orthologs from the phylogenetic tree was studied for GO and Pfam terms reviewed carotenoid biosynthetic gene products (Additional file 16: Table 4, Fig. 4). To further confirm the orthologs groups, a forest analysis (Fig. 5) was exploited as a “population analysis” using 47 well characterized *A. thaliana* reference sequences from TAIR (Additional file 17: Table 5) and the total *B. rapa* Pekinensis group population filtered (Additional file 14: Table 2, 386 elements); the analysis was performed to have a different and broader spectrum of sampling. In details, the 62 sequences from the “forest analysis”, the 44 elements of *B. rapa* Pekinensis group potential orthologs derived from the previous phylogeny and the 47 conserved carotenoid gene products from *A. thaliana* were subjected to a new phylogenetic analysis (Fig. 6**A**). The phylogenetic tree gave a result of 40 orthologs (see Additional file 18: Table 6).

All the aforementioned biased analysis allowed to specifically identify 40 not conserved carotenoid gene products in *B. rapa* Pekinensis group, but I were not sure about how many orthologs a single carotenoid element can have. Different *A. thaliana* proteins have more than one syntenic ortholog as also shown in previous finding [53]. I did not perform a syntenic analysis since I were not focused at the level of a pangenome architecture or genome assemblies, as the main purpose of this work has been a filtering of raw data. However, the high conservation among different protein clusters gave putative information about a possible syntenic relation (Fig. 6**A**). To verify the syntenic relationships I only suggest to run a Multiple Sequence Alignments (MSA) scanning algorithm if necessary [79, 80].

To look for putative syntenic clusters of proteins, two phylogenetic analyses were inferred by taking the elements belonging to the MEP pathway and the *GGPS* isoforms related (Fig. 6**B**, **C**). Here, three outgroups (Bkt - *β* carotene ketolase - from *C. reinhardtii*, Pds from *C. reinhardtii*, - Brp/brk *β*-carotene from *H. psychrophilus*), and one outgroup (hemoprotein cytochrome P450 - from *A. thaliana*) were included in the analysis in order to better estimate the divergence. The aforementioned species had some different evolution events in relation to the carorenoids pathway [31, 81] leading to a different regulation of carotenoid elements due to various adaptive fitness of the species (see Results). The *C. reinhardtii* element was selected as outgroup since it is an essential plant carotenoid biosynthetic enzyme. *H. psychrophilus* element was used as outgroup by retrieving the few information in literature databases [31, 42] (https://www.brenda-enzymes.org/enzyme.php?ecno=1.13.11.63onlyTable=Sequence; https://www.uniprot.org/uniprot/A0A1H3DR50). Furthermore, the hemeprotein cytochrome P450 from *A. thaliana* was used as an outgroup since it is working as monooxygenase for metabolizing various xenobiotic substances. P450 was used as a negative control because it is not a carotenoid biosynthetic gene product [31, 82].

First, I used various string searches queries via API linked to Uniprot-Swiss to apply different type of sampling and minimize the noise due to database redundancy and not univocal ID. Via different API (see Methods and Results) I retrieved different datasets of proteins in KOG, EggNOG, InParanoid, InterPRO, ProSite, EMBL, SUPFAM, RefSeq. The results of the last two phylogenic analysis (Fig. 6**B**, **C**) confirmed that a number of carotenoid elements were not found in *B. rapa* Pekinensis group and that some carotenoid biosynthetic gene products of *A. thaliana* correspond to one gene product in *B. rapa* Pekinensis group. Interestingly, *GGPS11* gene product was not found in *B. rapa* Pekinensis group, but a further Blastp query search (E-value around 80%) in NCBI coupled with a string search in EnsemblPlant database could get a putative correspondence unreviewed on EnsemblPlant. Interestingly, I found three putative conserved elements (Bra001832, Bra036950 and Bra008358 gene products) that need further investigation, via a syntenic analysis.

Despite of the presence of some “noise” due to the unbalanced classification of the databases, my pipeline generally confirmed the results previously found in *B. rapa* Pekinensis group [53]. The final findings of the present work are shown in Additional file 19: Table 7. It is worthwhile to mention that a sort of error occurs. This error, that I estimate as the percentage of extra putative orthologs not found in the reference work [53] with respect to all the orthologs found here, can be calculated as 5.6%. No comparisons can be made with similar methodologies, as my pipeline is a totally new method for applying deterministic filtering coupled with different phylogenetic analyses, starting with manually filtering then combining the filtered data and the more specific dataset retreived and finally implementing biased analyses. On the contrary, in previous findings, datasets are generally retrieved by using different type of sampling which mainly relies more or less on biased approaches [83–87]. Therefore, no previous similar pipeline was applied and no comparative study can be conducted with regard to error estimation, which should be done in comparison of the findings of the reference work that I took in consideration to evaluate the validity of my pipeline.

## Conclusions

**Fig. 7:**
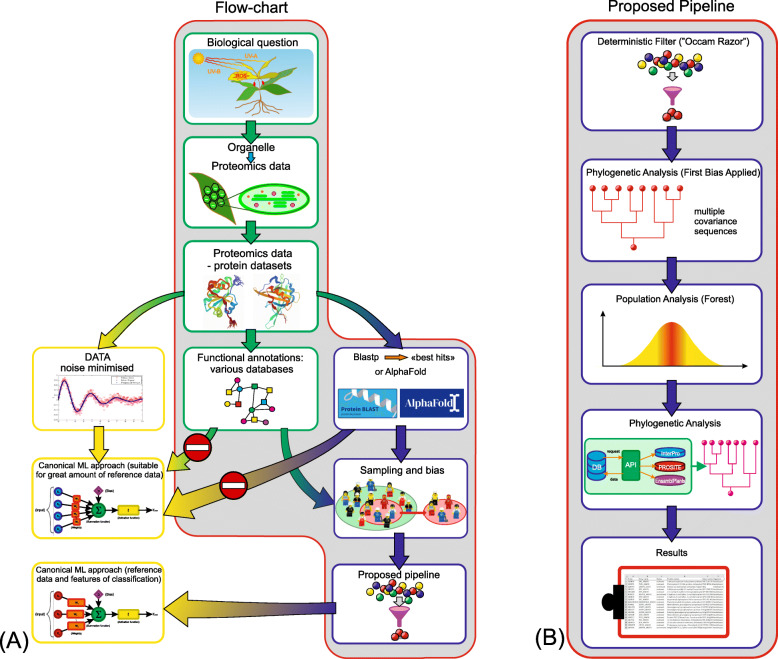
Model of bioinformatics pipeline method. **A** A method of filtering raw data could be applied to retrieve information at the level of families and superfamilies (domains, catalytic activities conserved motif). Deep learning is not always a feasible solution, because the signal-to-noise ratio must be high. Thus, it is necessary to preprocess the input data to minimize the noise, either selecting the best hits by the means of Blastp or performing an Alpha Fold 2 analysis. If neither of these processing is possible (mainly, due to the lack of univocal identification numbers reported in databases), I can preceded by filtering functional annotations in various databases to avoiding false positive weights in sampling and bias applied to the analysis. **B** In the latter case, the way to proceed is by the filtering pipeline shown here, which summarizes the investigation carried out by this paper

A filtering process is a helpful tool for screening and classifying information from proteomics studies (Fig. 7). Here I report the use of a pipeline for classification of protein orthologs for the study of carotenoid biosynthesis proteins in a group of species of commercial interest, the cabbages of the Pekinensis group.

A number of 45 carotenoid biosynthetic gene products was retrieved (see Additional file 19: Table 7). However, the table included the photosystem II element (*PBS*) and the *GPS11* which needed to be retrieved via Blast and Ensambl Plant searches. In conclusion, discarding the above mentioned elements (which would need further investigation), the retrieved orthologous are 43. The error of the analysis is estimated to be 5.6%.

The proposed tools are particularly useful when there is uncertainty about which factors are more relevant than others when classifying data (Fig. 7**A**). When a system is perturbed by the means of irrelevant datasets included in the analysis, I are tempted to apply wrong biased analysis which can lead to false-positive results (theory of chaos model, [88, 89]).

The proposed method could be applied when enough information are available at a level of family and superfamilies (domains, catalytic activities conserved motifs) about the proteins of interest and can be applied to different type of protein datasets. On the contrary, as shown in Fig. 7**B**, when dealing with lack of information, I suggest to perform a Blastp with restricted E-value selection to underline possible characterized proteins in the database or, if there is low similarity, I suggest to use Alpha Fold 2 algorithm [90] for a structure-based homology search [91].

## Methods

In this work I evaluated the efficiency and accuracy of a pipeline for protein classification in the two well-studied plants which belong to the Brassicaceae family: *A. thaliana* and *B. rapa* Pekinensis group. To notice, the different databases screened catalogue *B. rapa* Pekinensis group under different labels: *B. rapa* subsp. *pekinensis* and *B. rapa campestris*. Hence, the results presented in the tables keep the original labels of the database sources. A method of classification of raw data was evaluated to understand how different types of analysis can be applied depending on the sampling of the data in order to minimize the noise which can lead to a wrong bias and compromise the output results (false positive). The analysis has been compared with previous studies on carotenoid biosynthetic genes in *B. rapa* where phylogenetic analysis was computed and coupled with transcriptional profiles analysis [53].

I chose to screen libraries in public databases and avoid alignment-based techniques (i.e. BLAST – Basic Local Alignment Search Tool – [92], MAFFT – Multiple Alignment using Fast Fourier Transform – [93]) since sequences with low similarity among them are subjected to performance degradation and moreover this method could need a long processing time on large datasets. The computational proceeding includes the use of a “deterministic filtering” step, GO terms and Pfams screening for function and “population analysis” coupled with phylogenetic analysis.

I worked on the amino acid sequences which provide functional information (domain and motif) that is not straightforwardly visible in the nucleotide sequence.

### Flow of filters

The chloroplast and plastid biomes of *A. thaliana* were retrieved from the databases (Uniprot-Swiss/TrEMBL) where they were identified based on the proteomic studies. Additionally, the string searches related to the “photosynthetic process”: “chloroplast proteome”, “thylakoid proteome”, “carotenoids” were used.

Taken all the data together, 5222 proteins were retrieved. Panther and PFamCrossReference were also used as a collection of protein families compiled using multiple sequence alignments and hidden Markov models [94]. Pfam and GO information were saved in an excel file expanded to include available protein information in Uniprot - Swiss TrEMBL database and related API information linked. The same string searches were used for *B. rapa* Pekinensis group as well. Filtering steps carried out with a commercial software (MATLAB [95, 96]) were computed afterward (see Additional file 13: Table 1, Additional file 14: Table 2).

The final populations of *A. thaliana* and *B. rapa* Pekinensis group consist on 813 and 386 proteins respectively. The populations of the two species were fused and the redundancy of protein elements was checked via script with a result of 1089 total elements after the screening.

### *B. rapa* Pekinensis group phylogeny and gO terms of putative carotenoid orthologs

All the sequences obtained from *A. thaliana* and *B. rapa* Pekinensis group were aligned via Multiple Sequence Alignment Muscle-UPGMA method (hierarchical clustering) with the aid of a commercial software (MEGAX, [46]) and the phylogenetic analysis was subsequently inferred.

To build a phylogenetic tree I choose to apply a Maximum Likelihood (ML) approach. since this I believe that this method is a valid approach to parameter estimation problems and can be implemented for a broad variety of estimation situations. On the contrary, I decided to not use Neighbor Joining (NJ) and UPGMA (although I used the latter only for a prior alignment, as stated above) since this types of clustering algorithms, even if they can rapidly design cladrograms, have demonstrated a lack of reliability, particularly in cases of great divergence times. In particular, NJ was not applied because it is generally considered a phenetic method rather than a phylogenetic one. As a matter of fact, it uses the genetic distance (a phenetic criteria) between sequences to establish relationships, without considering any evolutionary model (ancestry) [97–100].

A phylogenetic tree was built with the following parameters:

1. the maximum likelihood [101] showed for 100 bootstraps to define the probability of the observed alignment occurring within 100 times.
2. the likelihood for clusters probability (*p*) for a series of 100 analysis which gives a lower variance than other methods; in this case, the variance of the distance (*d* – number of amino acid substitutions per site) is estimated by the bootstrap method.
3. the distance matrix used the JTT matrix (*F*) [102] and consists of the observed proportions of amino acid pairing between a pair of sequences where their divergence time (*t*) is given.
4. the gamma distribution in which the number of substitutions at each site were inferred using parsimony on the Bayesian estimates of the tree topologies [103].

The tree showed the clusters and allowed us to identify the putative conserved carotenoids in plants.

The tree with the highest log likelihood (-376192.95) was shown and it was drawn to scale, with branch lengths measured in the number of substitutions per site. This analysis involved 1089 amino acid sequences. All positions containing gaps and missing data were discarded (complete deletion option). A total of 34 positions resulted in the final dataset (Additional file 1). The percentage of trees in which the associated taxa clustered together is shown next to the branches. Initial tree(s) for the heuristic search were obtained automatically by applying Neighbor-Join and BioNJ algorithms [104] to a matrix of pairwise distances estimated using the JTT model, and then selecting the topology with superior log likelihood value. A discrete Gamma distribution was used to model evolutionary rate differences among sites (5 categories (+G, parameter = 0.0500)).

The list of orthologs from the phylogenetic analysis was studied for GO terms and Pfam terms (when available), retrieved from UniProt database (see Flow of filters): I screened them based on GO and Pfam terms and a heat map was used to visualize the GO-protein associations via scripts (Fig. 4, Additional file 2).

### Forest analysis coupled with phylogeny

To further confirm the orthology relationships, I computed a “population analysis” with the aid of a commercial software [95]. Following, the analysis was coupled by a phylogenetic tree. The population of the total screened *B. rapa* Pekinensis group (Additional file 14: Table 2, final filtering step) is evaluated by the means of the “MATLAB function localalign”. The higher is the score, the more correlated are the sequences. Each sequence of the population under test is evaluated against each sequence of the reference population (*A. thaliana* carotenoid sequenced retrieved from TAIR [105], Additional file 17: Table 5).

Given two sequences, the localalign algorithm developed by George Barton is efficient to locate all locally optimal alignments between two sequences allowing for gaps [96]. Localalign (SEQ1, SEQ2) (https://www.mathworks.com/help/bioinfo/ug/identifying-significant-features-and-classifying-protein-profiles.html) finds the optimal local alignment between two sequences, SEQ1 and SEQ2 (FASTA sequences) returning the highest-scoring local alignment and related information. To retrieve multiple local alignments, I limited the number of alignments by using the option NUMALN, MINSCORE. The sequences selected are those that have a score value greater than or equal to the threshold defined as:

$$\begin{aligned} ScoreThreshold = ScoreMean + 0.5\!~\times~(ScoreMax - ScoreMean); \end{aligned} $$

this value is halfway between the average and the maximum and it can be compared with two reference sequences. The score threshold reveals a bimodal distribution form with a first circumscribed region corresponding to the many cases in which the sequences correlate for a short duration with the samples. In a second much more diluted region, the sequences potentially well correlated with the sample population can be sought. Here, the reference sequences from *A. thaliana* were aligned versus the chloroplast proteins from *B. rapa* Pekinensis group. The result (Fig. 5) looks like a “forest” with some “trees” extending high over the “vegetation of the undergrowth”. As many sequences give very low score values, I have pruned to keep the mixFactor values separated in order to have more degrees of freedom. Sequences with high homology (score above 200) were used for the following analysis.

The evolutionary history was inferred according to the same protocol discussed in the previous section. The tree with the highest log likelihood (-38190.90) is shown (Fig. 6**A**). It is worthwhile to mention that the tree in this figure, as well as the trees in the following two figures, was elaborated with the commercial software iTOL (https://itol.embl.de/) and the bootstrap is reported in a scale 0 to 1. The nodes and the leaves of the tree are also presented in the tree, in order to indicate the different clusters. The suffixes “RA” and “ARA” for each protein ID sequence refer to *B. rapa* Pekinensis group and *A. thaliana*, respectively. For modeling evolutionary rate differences among sites, a discrete Gamma distribution was used (5 categories (+G, parameter = 5.4078)). The rate variation model allowed for some sites to be evolutionarily invariable ([+I], 0.00 sites). The analysis involved 122 amino acid sequences. All positions with less than 80% site coverage were eliminated, i.e., fewer than 20% alignment gaps, missing data, and ambiguous bases were allowed at any position (partial deletion option). Total of 270 positions resulted in the final dataset. Evolutionary analysis were conducted in commercial software MEGA X.

Furthermore, the phylogenetic analysis coupled with alignment was computed to confirm putative orthologs in the MEP pathways, *GGPP* gene pathway, and carotenoid biosynthesis.

### MEP pathway screening via phylogenetic analysis

To specifically retrieve all the potential syntenic and non-syntenic orthologs in *B. rapa*, *A. thaliana* reference carotenoids sequences and *B. rapa* Pekinensis group potential carotenoids elements were retrieved by using string searches in Uniprot database, EnsemblPlants, Inparanoid, Prosite, InterPro, KOG, Superfamily and STRING API linked to Uniprot. Here I wanted to checked more databases to retrieve all the possible information about the datasets related to the MEP pathways by combining two more selective phylogenetic analysis. In details, 35 elements from *B. rapa* Pekinensis group were retrieved from STRING and KOG using the string queries “oxidoreductase” and “reductase” and added to the 40 elements obtained from the Forest coupled with phylogeny. Following, the *B. rapa* Pekinensis group elements were added to the 49 sequences of *A. thaliana* putative carotenoids elements involved in the MEP pathway retrieved from Inparanoid, EnsemblPlants, InterPro, RefSeq databases via string queries “carotenoid” and “isoprenoids” (see Additional file 5). This implies that the deeper the divergence times, the more likely this method will lead to erroneous groupings. Therefore, I added different outgroups as controls to better test and estimate the divergence [106–108].

Indeed, four outgroups were selected in different organisms to improve the branch length of the subsequent phylogenetic analysis tree and the visualization of the clusters (see Results). Finally, the tree with the highest log likelihood (-71352.70) is presented in Fig. 6**B**. The topology of the tree with superior log likelihood value is selected. A discrete Gamma distribution was again applied like in the previous analysts: the evolutionary rate differences among sites (5 categories (+G, parameter = 2.0616)) and the rate variation model allowed for some sites to be evolutionarily invariable ([+I], 0.00% sites). All positions with less than 50% site coverage were discarded, i.e., fewer than 50% alignment gaps, missing data, and ambiguous bases were allowed at any position (partial deletion option).

For the *GGPS* elements another more detailed analysis was performed. Carotenoids elements from *B. rapa* subsp. *pekinensis* were retrieved by using the string search of “isoprenoids biosynthesis” for a total number of 37 KOG elements from EnsemblPlants, KOG, EggNOG, InParanoid, InterPro and 27 elements from EMBL and InterPro via the query “carotenoids oxygenase” from KOG, Inparanoid and Interpro. 17 carotenoids elements were retrieved from InParanoid, Prosite, EggNOG, SUPFAM, KEGG [109–111] and EMBL by using the string search “GGPS” for *A. thaliana*.

The four outgroups used in the MEP pathway tree analysis were added to the list above in order to obtain a more specific phylogenetic clustering computational method. The final dataset was subjected to phylogenetic analysis by using Maximum Likelihood method and JTT matrix-based model and the resulting tree with the highest log likelihood (-41791.08) is shown in Fig. 6**C**.

Initial tree for the heuristic search was obtained automatically by applying the Maximum Parsimony method. A discrete Gamma distribution was used for modeling evolutionary rate differences among sites (5 categories (+G, parameter = 2.1935)). The rate variation model permitted for some sites to be evolutionarily invariable ([+I], 0.00% sites). All positions with less than 50% site coverage were eliminated, i.e., fewer than 50% alignment gaps, missing data, and ambiguous bases were allowed at any position (partial deletion option) with a result of a total of 410 positions in the final dataset.

## Supplementary Information

**Additional file 1** phylognetic analysis between identifed elements and the carotenoid biosynthetic pathway.

**Additional file 2** Heat map showing potential chloroplast carotenoid orthologs.

**Additional file 3** Fasta file containing 62 protein sequences from B. rapa Pekinensis group that resulted from the forest analysis.

**Additional file 4** Elements from B. rapa Pekinensis group retrieved from different database sources. Entry IDs, protein names and the databases sources are highlighted in yellow.

**Additional file 5** Elements from A. thaliana retrieved from different databases: EnsemblPlants, InterPro, RefSeq and Inparanoid (highlighted in blue). The different columns present addional catalogued information retrieved via API linked to different Uniprot sources (highlighted in green). The identified proteins are putative carotenoid elements involved in the MEP pathway.

**Additional file 6** Elements from B. rapa pekinensis group retrieved from KOG, InParanoid and EnsemblPlant databases via the string search of “isoprenoids”. The column shows the different sources highlighted in blue. Some information are incomplete in the databases sources.

**Additional file 7** Elements from B. rapa pekinensis group retrieved from InParanoid, InterPro and EMBL databases via the string search of “carotenoid oxygenase”. The columns show the different sources and Entry IDs highlighted in blue. Additional information retrieved via API linked to Uniprot are also reported.

**Additional file 8** Elements from A. thaliana retrieved from Prosite, InParanoid, SUPFAM, EMBL and EggNOG databases via the string search of “GGPS” Additional information are retrieved via API from Uniprot linked to different databases sources. The columns show the different sources, Entry name and Protein families highlighted in blue.

**Additional file 9** script used to filter the population of A. thaliana and B. rapa - refer to Fig. 3A for A. thaliana and Figure 3B for B. rapa

**Additional file 10** Script used for generation of heatmap related to GOs and Pfam analysis - refer to Figure 4 and Additional file 2.

**Additional file 11** Script used for the forest analysis based on the gaussian fitting - refer to Fig. 5 and Additional file 12.

**Additional file 12** Flow-chart summarizing the whole process combining the forest analysis coupled with phylogeny and the phylogenetic analysis of the MEP pathway enzimes.

**Additional file 13** Steps of the filter population in A. thaliana.

**Additional file 14** Steps of the filter population in B. rapa Pekinensis group.

**Additional file 15** Table showing the discarding of the orthologs which did not play a role in oxygenic photosynthesis (labeled in yellow) resulting in 44 potential orthologs.

**Additional file 16** GO and Pfam terms reviewed Carotenoid biosynthetic gene products.

**Additional file 17** Characterized A. thaliana reference sequence from TAIR.

**Additional file 18** Orthologs resulting from the tree obtained from the phylogenetic analysis of 44 elements of B. rapa Pekinensis group and 47 conserved Carotenoid gene products from A. thaliana.

**Additional file 19** Table showing the Final finding of the whole process.

## Data Availability

The datasets analysed during the current study are available in the following repository: UniProt repository [https://www.uniprot.org/help/api] Pantherdb repository [http://www.pantherdb.org/] Protein Fam [http://pfam.xfam.org/] Arabidopsis repository [www.arabidopsis.org/] InParanoid [https://inparanoid.sbc.su.se/] Prosite [https://prosite.expasy.org/] KOG [https://www.ncbi.nlm.nih.gov/research/cog] KEGG [https://www.kegg.jp/kegg/kegg1.html] Ensembl Plant [https://plants.ensembl.org/index.html] EMBL-EBI [https://www.ebi.ac.uk/] NCBI [https://www.ncbi.nlm.nih.gov/] BRENDA [https://www.brenda-enzymes.org/] ncbi repositry [https://blast.ncbi.nlm.nih.gov/Blast.cgi/] The datasets used as input of the proposed classification method are also available as supplementary files. The resultant datasets, output of the proposed classification method, are available as excel file.

## Abbreviations

ABA: Abscisic acid
API: Application Programming Interface
ARA: *Arabidopsis thaliana*
BLAST: Basic Local Alignment Search Tool
BRARP: *B. rapa* subsp. *pekinensis*
BRACM: *B. rapa* L. (pekinensis group)
CNN: Convolutional Neural Network
EMBL EBI: European Molecular Biology Laboratory European Bioinformatics Institute
GGPS: GeranylGeranyl DiPhosphate
GO: Gene Ontology
JTT: Jones-Taylor-Thornton
KOG: Eukaryotic Orthologous Group
MAFFT: Multiple Alignment using Fast Fourier Transform
MEP: MethylErythritol Phosphate
ML: Maximum Likelihood
MSA: Multiple Sequence Alignments
NCBI: National Center for Biotechnology Information
NJ: Neighbor-Joining algorithm
NPQ: Non-Photochemical Quenching
Pfam: protein families
RA: Brassica rapa Chinese Cabbage Group
RF: Random Forest
ROS: Reactive Oxygen Species
TAIR: The *Arabidopsis* Information Resource
TrEMBL: Translated European Molecular Biology Laboratory
UPGMA: Unweighted Pair Group Method with Arithmetic mean
WGT: Whole Genome Triplications
