# Supplementary figures and images for: Developing a bioinformatics pipeline for comparative protein classification analysis
Source: BMC Genom Data. 2022 Jun 6;23:43. doi: 10.1186/s12863-022-01045-x (PMC9172112; doi:10.1186/s12863-022-01045-x)

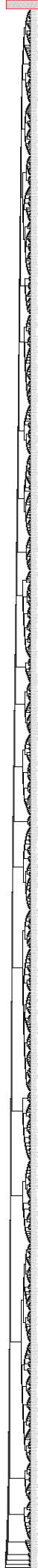

Supplement: Supplementary file 1 — Additional file 1 phylognetic analysis between identifed elements and the carotenoid biosynthetic pathway. [file 12863_2022_1045_MOESM1_ESM.pdf]

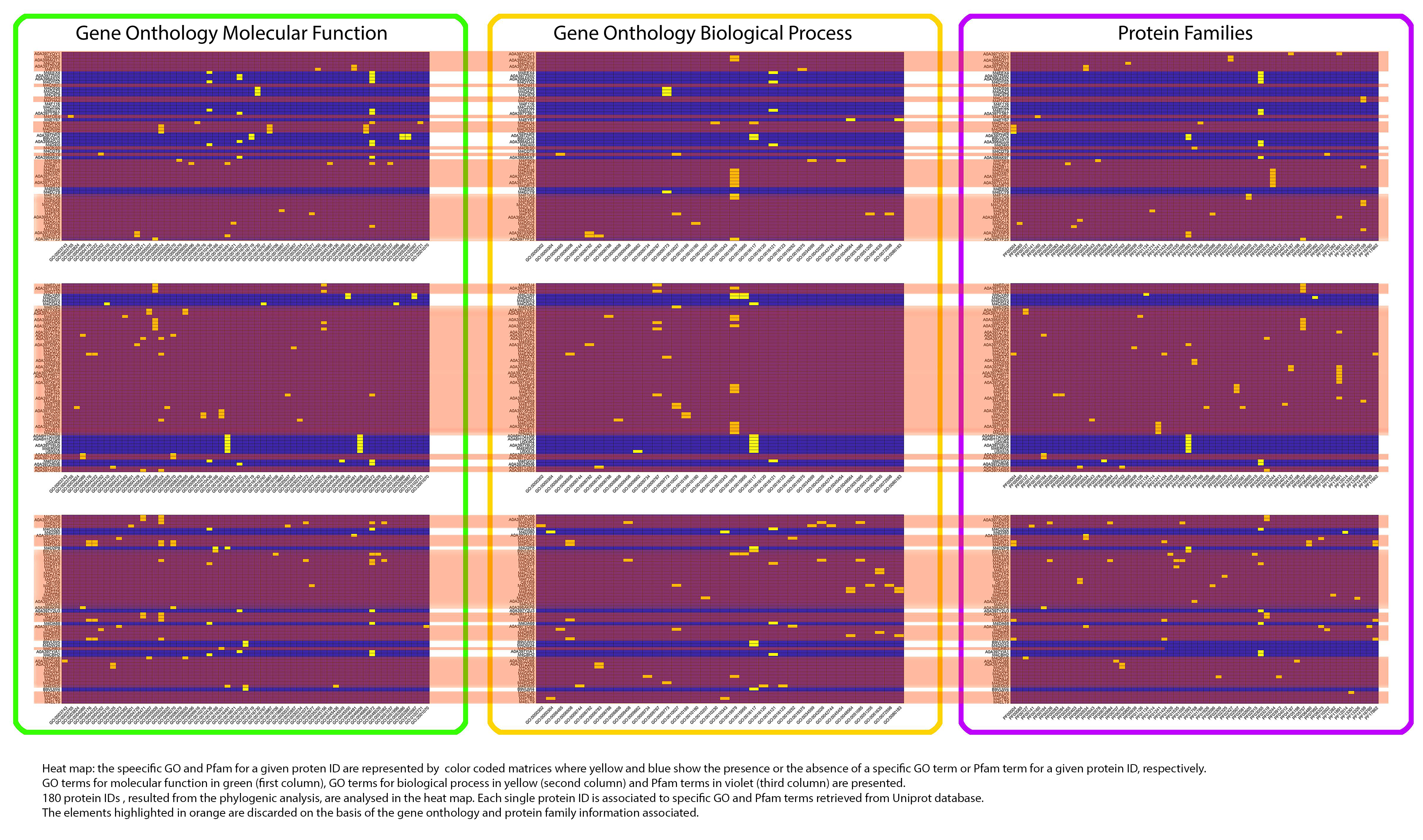

Supplement: Supplementary file 2 — Additional file 2 Heat map showing potential chloroplast carotenoid orthologs. [file 12863_2022_1045_MOESM2_ESM.png]

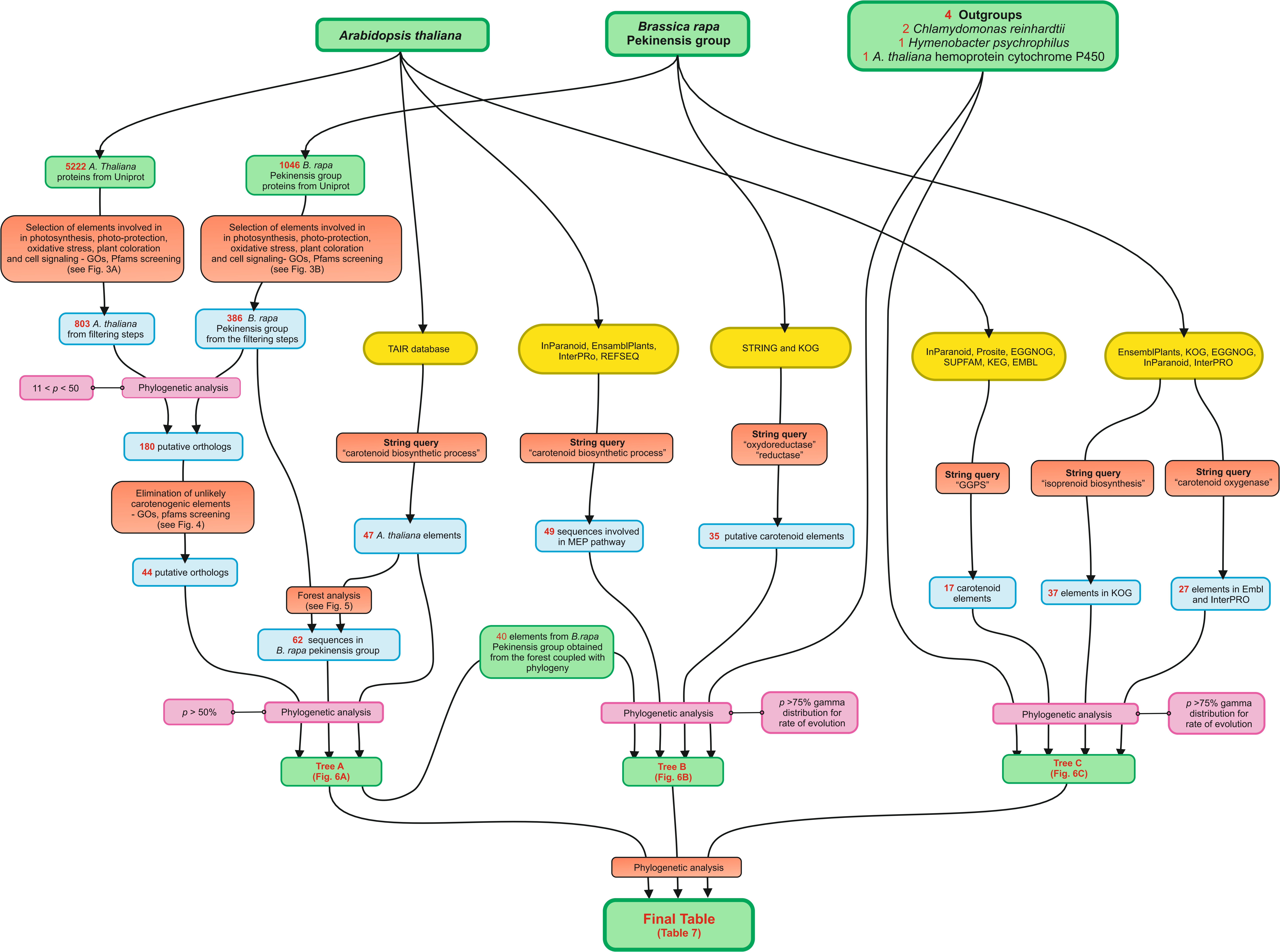

Supplement: Supplementary file 12 — Additional file 12 Flow-chart summarizing the whole process combining the forest analysis coupled with phylogeny and the phylogenetic analysis of the MEP pathway enzimes. [file 12863_2022_1045_MOESM12_ESM.pdf]
